# Environmental Risk Factors for Developing Type 2 Diabetes Mellitus: A Systematic Review

**DOI:** 10.3390/ijerph15010078

**Published:** 2018-01-05

**Authors:** Tashi Dendup, Xiaoqi Feng, Stephanie Clingan, Thomas Astell-Burt

**Affiliations:** 1 Population Wellbeing and Environment Research Lab (PowerLab), School of Health and Society, Faculty of Social Sciences, University of Wollongong, Wollongong, NSW 2522, Australia; xfeng@uow.edu.au (X.F.); stc51@uowmail.edu.au (S.C.); 2 Menzies Centre for Health Policy, University of Sydney, Sydney, NSW 2006, Australia

**Keywords:** type 2 diabetes mellitus, environment, walkability, green space, noise, air pollution

## Abstract

Different elements of the environment have been posited to influence type 2 diabetes mellitus (T2DM). This systematic review summarizes evidence on the environmental determinants of T2DM identified in four databases. It proposes a theoretical framework illustrating the link between environment and T2DM, and briefly discusses some methodological challenges and potential solutions, and opportunities for future research. Walkability, air pollution, food and physical activity environment and roadways proximity were the most common environmental characteristics studied. Of the more than 200 reported and extracted relationships assessed in 60 studies, 82 showed significant association in the expected direction. In general, higher levels of walkability and green space were associated with lower T2DM risk, while increased levels of noise and air pollution were associated with greater risk. Current evidence is limited in terms of volume and study quality prohibiting causal inferences. However, the evidence suggests that environmental characteristics may influence T2DM prevention, and also provides a reasonable basis for further investigation with better quality data and longitudinal studies with policy-relevant environmental measures. This pursuit of better evidence is critical to support health-orientated urban design and city planning.

## 1. Introduction

The burden of diabetes is rising rapidly worldwide posing an enormous socioeconomic and health challenge [1,2,3]. The number of people with diabetes is estimated to further increase from that of 415 million in 2015 to 642 million by 2040 [2]. Type 2 diabetes mellitus (T2DM) characterised by excess blood sugar levels accounts for around 90% of the cases [2]. T2DM can cause severe damage to body systems such as kidneys, eyes, and the heart, as well as the vascular system more generally. The escalating burden of T2DM indicates that past prevention efforts via interventions designed to increase physical activity and promote healthy diet have not led to population-level gains. The plausible influence of neighbourhood and environmental characteristics on health including T2DM is increasingly being recognised and studied in the recent years [4,5,6]. However, no study has critically reviewed studies of the association between environment and T2DM risk.

This systematic review aimed to evaluate the literature on the environmental determinants of T2DM risk. It provides a brief summary of the natural history of T2DM followed by a conceptual framework illustrating the possible links between the environment and T2DM. Further, some methodological challenges in studying the environment and potential strategies to overcome them are discussed. After that, a critical synthesis of the existing literature on the environment–T2DM relationship is presented. The subsequent section discusses our findings and also highlights future research directions. 

## 2. Biology of T2DM

A person living with T2DM does not produce enough insulin (insulin deficiency), or has body cells that are not able to use insulin properly (insulin resistance) [7]. Insulin, a hormone produced by the β-cells in the pancreas, controls blood sugar levels. Insulin resistance is related to genetic factors [7,8], obesity, sedentary lifestyle and aging [7,9]. Consumption of energy-dense food and physical inactivity are important predictors of obesity and T2DM [9,10].

Initially, a greater amount of insulin is produced to achieve a normal glucose level [11,12]. However, this response is inadequate to overcome insulin insensitivity particularly in obese individuals contributing to an increased production of glucose by the liver [11]. This leads to “prediabetes” condition, wherein the glucose levels are high but under the T2DM range. The metabolism of carbohydrate, fat, and protein are disturbed as the disease progresses [7,13]. Hyperglycaemia (high blood sugar levels) results when the β-cells fail to compensate insulin resistance with excess insulin output [12]. The progressive decline of the β-cell function and mass over time with hyperglycaemia marks the development of T2DM [11,13].

Accumulation of fat in the liver, muscles, and pancreas from surplus calories and physical inactivity contributes to β-cell dysfunction and insulin resistance [12]. Inflammation, oxidative and endoplasmic-reticulum stress, raised lipid levels, and amyloid accumulation also trigger β-cell dysfunction [11,13,14]. Gastrointestinal tract hormones and nervous system including the brain also acts on β-cells and glucose metabolism [12,13]. Early diagnosis and treatment with lifestyle interventions (physical activity, diet, and weight loss) and glucose-lowering medications can reduce complications and vascular diseases, and prevent or delay disease progression [7,9,13,14].

## 3. Mechanisms Linking Environment and T2DM

T2DM is hypothesised to be an outcome of the interaction of environmental, biological, and behavioural risk factors [13,15]. Healthy lifestyles are thought to be discouraged in the absence of an environment that supports them, and behavioural and educational interventions may be significantly diminished or rendered ineffective in such non-enabling environment. The evidence in the literature shows that individual-level socioeconomic, demographic, and behavioural factors are important predictors of T2DM [16,17]. Prior reviews also suggest a link between the environment and health outcomes closely related to T2DM such as obesity, cardiovascular diseases, hypertension, metabolic syndrome and physical activity [5,6,18,19,20,21,22,23]. 

**Figure 1:**
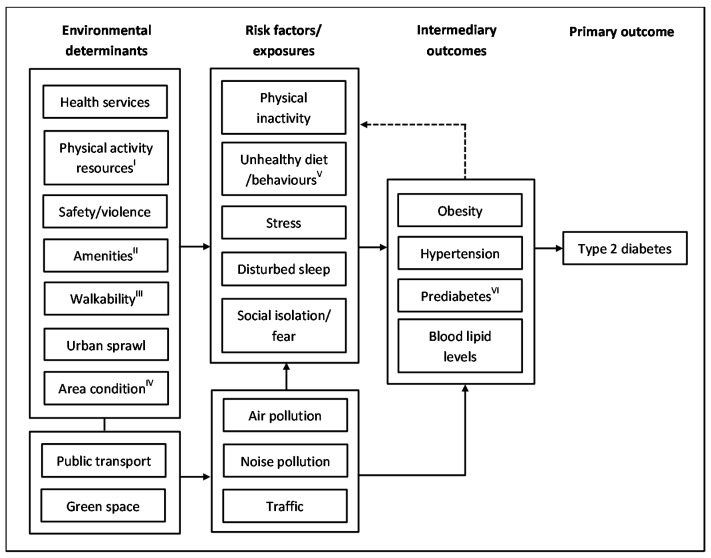
Schematic illustration of possible pathways through which environment impacts type 2 diabetes mellitus (T2DM) risk. Adapted from Poortinga [34], Northridge et al. [41], and Giles-Corti et al. [42]. ^I^ include walking, jogging and cycling infrastructure, open spaces, trails, etc.; ^II^ include supermarkets, shops, food outlets, recreational and other facilities, etc.; ^III^ include street connectivity, density, land use, sidewalks, walkable destinations, etc.; ^IV^ include housing condition, design, aesthetics, etc.; ^V^ smoking and drinking; ^VI^ high blood sugar levels below the range of T2DM diagnosis, also referred to as impaired glucose tolerance (IGT) or impaired fasting glucose (IFG).

The conceptual framework presented in Figure 1 illustrates the possible pathways through which different characteristics of the environment may determine T2DM. This framework is underpinned by socio-ecological theories that emphasise human behaviour is influenced by their ability, and when their sociodemographic, psychosocial, economic, organizational and physical environment are supportive [24]. The framework also draws on the knowledge reviewed in this paper.

Environmental characteristics are hypothesised to increase exposure to risk factors of T2DM by enhancing or constraining behavioural, psychosocial and physical stressors. The physical and social environment can influence choices and behaviours [25,26]. Availability and/or proximity to recreational resources, green spaces, open spaces, walkable destinations, sidewalks, and well-designed and connected public places, higher land use mix can encourage physical activity and social interaction [6,21,23,27,28,29,30,31,32,33,34]. Individuals living in a highly walkable environment are likely to walk more [29,35], thereby reducing the risk of obesity. Similarly, having supermarkets close by can encourage a healthy diet, and dense neighbourhoods can facilitate access and use of local amenities, social activities [36,37,38], and physical activity [23,35,39,40]. In contrast, limited access to supermarkets may motivate visits to convenience stores and fast-food outlets that in turn increase the odds of unhealthy food intake. These environment shaped choices and behaviours can regulate calorie intake and burning that influences obesity risk, β-cell dysfunction and insulin resistance. 

Crime, social disorders, and unsafe neighbourhood may incite social isolation and fear [43] and inhibit physical activity [44], whereas strong social networks, safety, green space and pleasant scenery in a neighbourhood can improve mental health or counter related adverse effects [28,45,46] and encourage physical activity [30,34,39]. Social activities can also be diminished in sprawling areas owing to heavy reliance on cars and more travel time. Availability of social support and community resources and establishment of positive social norms through social interactions and networks can enable healthy choices and behaviours. A dense neighbourhood, however, may also increase stress and disorders [38] and promote unhealthy behaviours [6]. Prolonged exposure to multiple adverse environmental stressors can lead to “allostatic load” or the biological wear and tear of the body physiological systems [47,48]. The strain accrued from stress can stimulate the release of substances (such as cortisol and cytokines) that can damage the immune and body systems accelerating the development and progression of chronic diseases including T2DM [48]. Stress can also motivate unhealthy eating, smoking, and drinking, and affect sleep. Furthermore, access to unhealthy food environment may have a synergistic effect. These unhealthy behaviours and poor mental health can impact metabolic changes and body weight, increasing the risk of T2DM.

Air pollution has been documented to change endothelial function, trigger inflammation and insulin resistance, and is associated with elevated risk of hypertension [6,49]. Air pollution and road traffic noise may also adversely affect blood lipid levels [50,51] that in turn may influence blood pressure and T2DM risk. Some evidence suggests green space [52], transport system and traffic [30] may influence local pollution levels and physical activity. Air pollution may discourage exercise, while noise can affect sleep and mental well-being. Further, individual-level socioeconomic and related characteristics can also influence the effect of environment on T2DM. For instance, those with low income may be more vulnerable to adverse environmental conditions. Lastly, the determinants and progression to T2DM can be shaped by circumstances and changes throughout the life course. 

## 4. Methodological Issues in Studying Environmental Characteristics

### 4.1. Measurement and Misclassification of Exposures

Metrics such as density, diversity and distance are often used to assess environment and health. The availability of data and feasibility often determines their use and currently, it appears that there are no agreed standard metrics to measure specific environmental characteristics [18]. More recently, the use of Geographic Information System (GIS) has helped to overcome measurement bias from self-reported measures [4]. However, measures using GIS and other tools tend to ignore qualitative aspects such as cost, use, and quality. Defining and operationalising the spatial scope of the environment has also been a persisting issue [5,53,54]. Pre-defined areas such as census tract and blocks may not reflect the recent settlement patterns and residents’ perceptions. Neighbourhood can also be defined by social networks, transportation [53] and may differ by health outcomes assessed [55]. Further, small buffer areas may not capture distant factors, whereas population defined areas may encompass different areas in different geographic areas [4,18].

“Same-source bias”, which arises when both the outcome and exposure are self-reported within the same survey [54,55], can generate biased relationships. For instance, physically inactive persons may be less likely to report physical activity resources. The use of composite indices such as walkability can also present issues concerning validity, reliability, and generalizability [18]. Such indices may not also be beneficial in targeting interventions given the difficulty in identifying specific components and can be of little use in discerning underlying mechanisms. 

### 4.2. Confounding and Health-Selective Migration

Self-selection of individuals into neighbourhoods based on their health and predisposition to certain behaviours can lead to a spurious pattern that exaggerates or induces what could otherwise appear to be an environmental effect [56,57]. Health conscious individuals, for example, may choose to live in areas with better access to physical activity and healthy food resources [57]. Food outlets and recreational facilities can also be established depending on the neighbourhood demand [53,57]. Study results can be biased from reverse causation if these dynamics are not considered. Sociodemographic and economic characteristics including knowledge and attitudes regarding health and environment can influence the environment-health relationship. Factors such as education, age, and income can also determine an individual’s choice of place to live [56,58]. The ability of a study to infer valid findings and untangle the relationship is restricted if important variables are omitted and/or mismeasured [4]. 

### 4.3. Sampling and Secondary Data

Using data on individual-level characteristics from observational studies that are linked to area-level data can lead to the possibility of too few participants in a particular neighbourhood limiting the ability of studies to separate the individual and neighbourhood effect [56]. In addition, the failure to include important variables will prevent statistical adjustment, and provide less information on the environment–T2DM pathway. Finally, data from databases may not be spatially accurate or not reflect the situation during the study period in question.

## 5. Identification Strategies

Stratification and regression methods including multi-level approaches are widely used to adjust for individual-level characteristics [55]. Multilevel analysis also allows for exploration of individual and area variations separately that help determine the causal role of area characteristics [59]. These techniques are not able to account for omitted and mismeasured variables. Propensity score matching that limits the comparison to participants by balancing confounders among those exposed and unexposed can allow for better adjustment of these characteristics [60]. Instrumental variables estimation method that manipulates the exposure by identifying variables can also adjust for both measured and unmeasured confounders [61]. Longitudinal study designs allow assessment of temporal association potentially also accounting for selective migration [18,54]. Including larger areas and factors that determine the place to live may also help overcome self-selection issue [18,58]. Longitudinal data also permit assessment of cumulative effect, and exposure duration effect and changes in characteristics. Application of GIS and sensitivity analysis provides the opportunity to better define and identify the neighbourhood context. 

Ludwig et al. [62] employed a randomised intervention design to assess the influence of neighbourhood conditions on obesity and T2DM. Randomised experimental studies help eliminate confounding by known and unknown factors. It may not be nonetheless feasible and even ethical to conduct such studies given the difficulty to randomly assign individuals to different environment and also policies are implemented in real settings on a huge scale [4,5]. The requisite for many neighbourhoods and incomplete understanding of interventions to be tested also renders experimental study difficult [4]. Natural or quasi-experimental designs comparing those exposed and unexposed to environmental changes, and policy evaluation studies can help inform causal inferences [63,64]. Further, the meticulous application of Bradford Hill’s criteria can be useful in evaluating hypothesis [65]. 

Combining self-reported responses of several participants from the same neighbourhood for an exposure [55], and including participants not part of the outcome assessed [54], may help circumvent the “same-source bias” issue. The inclusion of an adequate number of participants using neighbourhood-based sampling and using both aggregated and individual data can mitigate the problem of inability to distinguish individual and contextual effect when linked individual-level and neighbourhood data used [56]. Collection of new and the use of most recent data are also essential to generate robust evidence. 

## 6. Methods

### 6.1. Search Strategy

A literature search using four electronic databases (PubMed, Web of Science, Science Direct, and Scopus) was conducted in April 2017. The keywords in Table 1 were searched in the titles and abstracts of the articles. Terminologies from other reviews and those suggested by the review team members were incorporated. The references of related publications were also searched. 

### 6.2. Eligibility Criteria

The inclusion criteria were: (1) quantitative studies reporting epidemiological data; (2) investigated at least one environmental characteristic as a main variable and assessed its association with T2DM and/or prediabetes i.e., impaired fasting glucose (IFG) and impaired glucose tolerance (IGT); (3) published in English; (4) participants were ≥18 years; (5) used objective and/or subjective environment measures; and (6) journal articles published since 2000. Studies that did not specify diabetes type were also included given that a majority (~90%) are of T2DM [2]. Studies on type 1 diabetes and gestational diabetes, and non-peer reviewed articles, commentaries, case reports and conference papers were excluded.

The environment in this review is referred to physical environmental surroundings changed by human activities that include sidewalks, schools, homes, parks, green space, highways, recreational facilities and amenities, roadways, etc. that can influence lifestyle and health [66,67]. This definition focuses upon, but is not limited to features of the built environment (e.g., air pollution). The environmental characteristics measured using physical observation, audits and GIS were categorised as objective measures, while those features measured through interviews and questionnaires were regarded as subjective measures [68,69].

### 6.3. Selection Strategy and Data Collection

**Figure 2:**
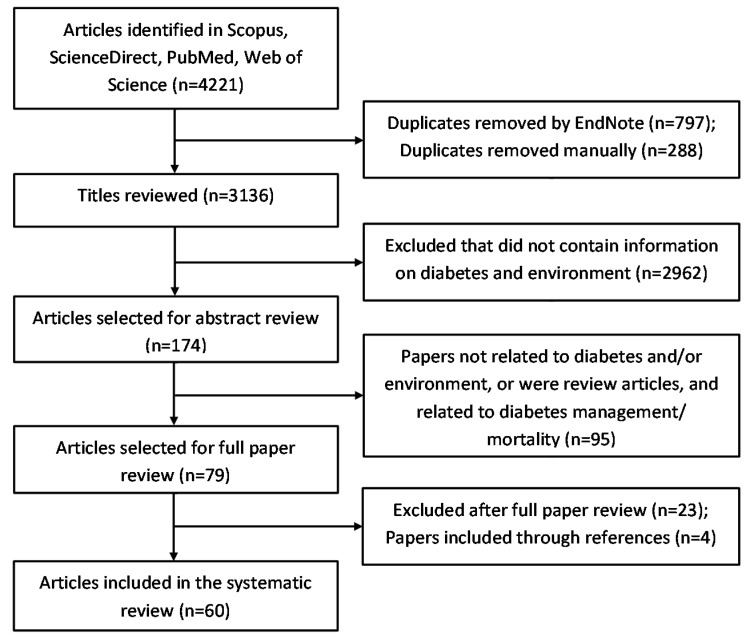
Flow chart illustrating the search and selection process.

Figure 2 illustrates the process to search and select articles. All articles were downloaded onto the reference manager EndNote version 7. Two reviewers independently examined the titles against the selection criteria followed by abstract review. Those studies requiring full-text assessment were reviewed again by both reviewers for the final selection. Discrepancies were resolved through discussion and consultation with a third reviewer.

### 6.4. Data Analysis

The data extracted from all selected articles are summarised in Table S1. The effect sizes of fully adjusted models (where available) along with their interval estimates are presented. The National Institutes of Health’s Quality Assessment Tool for Observational Cohort and Cross-Sectional Studies was employed to assess the study quality and risk of bias [70]. The evidence in the body of selected studies was narratively synthesised.

## 7. Results

**Figure 3:**
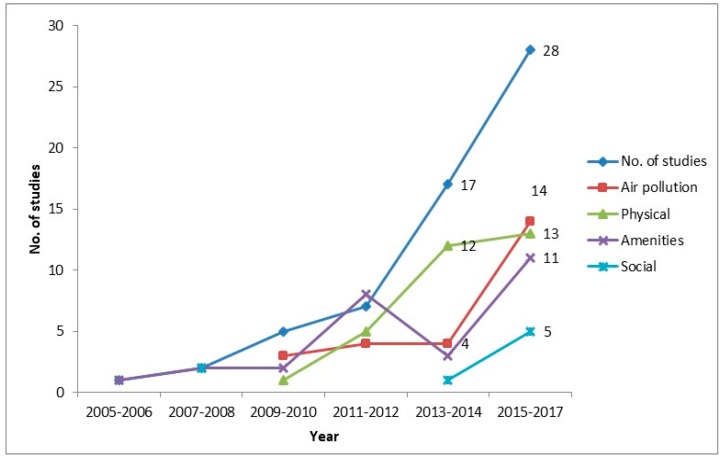
Number of studies and environmental characteristics studied by year. Physical includes roadways proximity, walkability, open space, green space, tree canopy, traffic, noise, urban sprawling, and slope; Amenities includes food and physical activity environment, recreational facilities, transport system, and health service; Social includes safety, violence, crime, physical disorder, area and housing conditions, and infrastructure.

Of the total 4221 articles, 1085 duplicates were removed (Figure 2). Upon reviewing the titles and abstracts, 3057 were excluded, leaving 79 articles for full review. A total of 60 studies were selected and assessed for the review. An overview of the assessed studies is provided in Table 2. The number of studies increased drastically in recent years (Figure 3). Almost all of the studies were from high-income countries. There were almost equal number of cross-sectional and cohort studies and there were no experimental studies. Many studies used secondary data from studies designed for other purposes and linked it with area-level environmental characteristics.

T2DM diagnosis was based on self-report in many studies (Table 2). Those with prediabetes were included under T2DM in four studies, and one [71] of them assessed the effect for T2DM and prediabetes separately. Three studies used survey or interviews and another six used a combination measure (GIS and/or database and surveys) to assess environmental characteristics. The most common environmental characteristics assessed were air pollution, food environment, physical activity resources, walkability, and roadway proximity. There was only one study that investigated urban sprawl, area-level slope, and availability of general practitioners. A little over half of the studies were of fair quality while 18% were of good quality. Many of the studies rated as good quality focused on air pollution. 

Of the more than 200 relationships reported and extracted, 82 showed significant association in the expected direction (Table 2). Several studies including younger adults (<30 years of age) showed no strong or no statistically significant results [72,73,74,75,76,77,78]. Most [73,79,80,81,82,83,84,85], but not all [86,87,88,89], studies conducted among ethnic groups and women also showed stronger associations. This trend was mainly found in studies that assessed air pollution. The findings for specific environmental characteristics in relation to T2DM risk are summarised in the subsequent paragraphs.

### 7.1. Walkability

Walkability is defined as the degree to which an area is conducive and supportive for walking [90]. It is expressed in terms of density, land use mix, design, connectedness, distance and destination accessibility, and safety and aesthetics often are also included. Almost all studies [85,91,92,93,94,95] applied a walkability index using at least three or more of such attributes. One study used physical observation in addition to GIS measure [96].

One study [91] reported a one standard deviation increase in walkability attenuated the risk of T2DM by 12%. Another two cohort studies also found significantly increased T2DM risk among those living in the least walkable environment [85,95], and one noted a slightly elevated effect among women, long-term residents, and recent immigrants [85]. The follow-up period was 3.5 years in the former and five years in the latter study. Further, the former study did not account for changes in environment and residents, while the latter study lacked information on BMI, physical activity, ethnicity, and food environment. A time series study also found similar significant protective effect of area walkability on T2DM [93]. 

Similarly, a higher level of walkability was found to be associated with lower T2DM risk in a cross-sectional [96] and an ecological study [94]. Another study found a non-significant lower T2DM (self-reported) probability among those living in the most walkable areas [92]. The effect appeared to be stronger among men and was significant when not controlled for physical activity and sedentary behaviour. One study [95] noted no difference among co-siblings living in areas with different walkability levels and the association lost significance when adjusted for sociodemographic factors. This study did not capture those likely people not on medication and could have underestimated the effect. Booth et al. [85] observed significant interaction between income and walkability with higher risk among low-income groups. Many studies did not account for safety, traffic, noise, pollution, crime and food environment [85,92,94,95,96].

Two studies assessed the impact of individual components of walkability. Only land use mix retained significance in one study [95], whereas all components were significant in the other [94]. The latter study, however, did not adjust for socioeconomic and demographic factors. Urban compactness, a measure correlated with walkability, was shown to be linked with lower T2DM prevalence in an ecological study [97]. 

### 7.2. Physical Activity Resources

Of the seven studies, two used survey measure [98,99], one used interviewer assessment [77], two used a combination of survey and GIS measure [73,100], and another two used information from databases [101,102] to assess physical activity resources. A cohort study using a combined measure (GIS and survey) found a reduction in T2DM risk by 19% for an interquartile increase in physical activity resources [100]. Stronger effect was evident when survey-based measure was used. A non-significant reduction in T2DM risk with increase in physical activity resources was observed in another cohort study [99], and a cross-sectional study that used survey-based measure [98]. A few other studies also displayed no significant association for availability and distance to recreational and physical activity resources [73,77,101,102]. Auchincloss et al. [99] noted a significant decrease in T2DM risk when the combined measure of physical activity and healthy food was used and the effect was significant when not accounted for BMI. This study did not find significant difference in risk when assessed by duration lived in the neighbourhood. 

### 7.3. Food Environment

Most studies applied presence and proximity to food environment within an area measured using GIS, information from databases, business listings, and surveys. A few studies used a ratio or an index of healthy or unhealthy food environment [91,103,104]. None of the studies accounted for exposure to food environment outside of neighbourhood such as workplace, and access to farmers and to fruit and vegetable markets were assessed only in a few.

A cohort study that interviewed participants showed that the risk of T2DM decreased by 37% among those with highest than those with least access to healthy food environment [99]. Mezuk et al. [103] using unhealthy to total food outlets ratio, reported that gaining access to more unhealthy food either in the same area or by locating to a new place was associated with a higher T2DM risk. This study did not control for physical activity, ethnicity, BMI, and found area socioeconomic status (SES) still significant in the final model. Another study found higher T2DM risk among African-Americans to be associated with higher density of unfavourable but not for favourable food stores [73]. Christine et al. [100] using a combination of GIS and survey-based measure, and GIS alone measure for healthy food environment also did not find a significant association. However, the effect was significant when survey-based food measure was used. Two studies also found no significant association between T2DM and unhealthy food outlets [74,91]. Physical activity, diet, area-level SES and BMI appeared to attenuate the T2DM risk [74,98,100]. 

A few cross-sectional studies did not find strong association between food environment and T2DM [98,105,106]. A study showed a significant marginal association between screen-detected T2DM and a higher number of fast-food outlets [107]. This study did not assess by fast-food outlets type and did not adjust for smoking, alcohol use and BMI. Studies showed no difference in T2DM risk when evaluated by sub-types within the unhealthy food category [104] and when they accounted for duration lived in the neighbourhood and relocation [100]. Frankenfeld et al. [104] showed significantly lower T2DM prevalence in areas with greater restaurants and speciality food than grocery store within healthier food options category. The effect of food environment was not evident in a few ecological studies [75,76,108], whereas another two showed mixed findings that used several different measures [101,102]. 

### 7.4. Green Space

All three cross-sectional studies found that greener neighbourhoods were associated with lower T2DM risk [72,109,110]. This protective effect of green space appeared to be smaller when a longer buffer radius was used to ascertain green space exposure [72], and more pronounced when an objective T2DM measure was analysed [110]. Astell-Burt et al. [109] reported a potential threshold effect, with the largest benefit for T2DM prevention observed among participants living in areas with 40–60% green space land-use. In addition, this study also reported similar degrees of benefit from green space, regardless of neighbourhood socioeconomic circumstances [109]. All three studies focused on the quantity, rather than quality of green space, which could also potentially play an important role. 

A related study found a non-significant lower probability of T2DM with more tree-canopy [111]. Likewise, a cohort study found no strong association between access to open space greenness and T2DM risk [91]. Public open space greenness and tree canopy may not capture the actual and total greenness in the neighbourhood that may be relevant for T2DM prevention. 

### 7.5. Residential Noise, Traffic, and Proximity to Roads

A few studies found a greater risk of T2DM among those exposed to higher noise levels [112,113,114]. A large cohort study found a significant increase in incident T2DM by 8% and 11% with an increase in 10 dB levels of road traffic noise at current residence and during five years preceding T2DM diagnosis, respectively [112]. Similarly, another cohort study showed that the T2DM risk increased by 35% with an inter quartile range (IQR) increase in road noise exposure [113]. Stronger effects were observed among confirmed T2DM, women, those with low education [112], and those who slept with open windows and reported poor sleep quality [113]. Sorensen et al. [112] also found no significant change in risk upon adjustment for NO_x_ (nitrogen oxides) suggesting an independent effect of noise. In contrast, railway [112,113] and aircraft noise [71,113] were not strongly associated with T2DM though the effect of aircraft noise was evident during daytime [113]. The effect of noise significantly differed by physical activity levels and sex but not by sleep quality [71]. Small sample size, subjective outcome measure, and non-random sampling [114], trivial cases and non-adjustment for other noise sources [71], and non-adjustment for bedroom location, other noise sources, and hearing impairment [112] were noted in some studies. 

With regard to traffic exposure, Heidemann et al. [115] demonstrated a higher T2DM risk among those whose homes were located at extremely busy through road compared to those whose homes were located at a street with no or rare traffic, but not for moderate, considerable and heavy levels of traffic exposure. Residential traffic noise appeared to mediate the relationship, and the effect was not altered when accounted for education, indoor air pollution and other factors [115]. A cohort study also showed a marginal significant association for traffic load within 100 m among confirmed T2DM cases [116]. Whereas one cross-sectional study found no strong differences [117], two found a non-significant association for high self-reported [114] and GIS measured traffic intensity [118].

A cohort study of health professionals found a greater T2DM risk among individuals living within 0–49 m than those living ≥200 m from the proximate road, but not for 50–199 m [119]. Kramer et al. [82] found a significantly higher risk of T2DM among those women living <100 m from a busy road with low education but not among those with high education. Likewise, another study also showed a significant higher risk of self-reported T2DM among those living close to a major road [120]. Several other studies, however, found non-significant or no differences in T2DM risk [86,116,117,118,121].

### 7.6. Air Pollution 

The most common pollutants examined in relation to T2DM were PM (particulate matters) and NO (nitrogen oxides). PM_2.5_ (particulate matter <2.5 μm in diameter), NO_2_ (nitrogen dioxide), PM_10_ (particulate matter <10 μm in diameter), and NO_x_ levels were in assessed in 14, 11, 8 and 5 studies, respectively. Several cohort studies showed greater T2DM risk to be associated with exposure to higher levels of NO [82,83,116], and PM_2.5_ [81,84,122]. Andersen et al. [116] using mean NO_2_ exposure measured by AirGIS at different point of time found a marginally significant increased T2DM risk for an IQR increase in NO_2_ among confirmed T2DM cases, but not for all T2DM cases and baseline NO_2_ levels. Another study also showed similar heightened T2DM risk from exposure to higher traffic-related PM and NO_2_ levels (from emission inventory) [82]. This study was limited by differences in some characteristics at baseline, small sample size, inclusion of older age group (54–55 years) and non-inclusion of SES. 

A study found higher levels of NO_x_ to be significantly associated with increasing T2DM risk after controlling for socioeconomic and anthropometric factors, but not for PM_2.5_ [83]. The effect of PM_2.5_ diminished when both pollutants were assessed together. Stronger effect was also evident for NO_2_ exposure in a study [82], and another noted that the effect of Ozone lost significance when controlled for NO_2_ but not when adjusted for PM_2.5_ [79]. Coogan et al. [88] also found that the addition of Ozone and PM_2.5_ did not alter the NO_2_–T2DM effect. Studies conducted among women showed exposure to greater levels of Ozone [79] and Soot [82] to be associated with elevated T2DM risk.

Puett et al. [119] using two cohorts of health workers when combined and considered separately, and when pollutants were modelled together and separately, did not find a significant T2DM risk among those exposed to PM_2.5_, PM_10_, PM_10–2.5_ (particulate matter 2.5–10 μm in diameter). This study lacked information on NO and had a small men sample. The non-significant effect of PM_2.5_ found in Coogan et al. [83] was corroborated in a follow-up study [89]. Similarly, the adjusted results in several studies suggests no strong or not significant association for exposure to NO_2_ [81,88,113], PM_2.5_ [83,113,119,120,121], NO_x_ [81,121], PM_10_ [81,82,119,120], and PM_10–2.5_ [119]. 

Eze et al. [123] using a cross-sectional data revealed a higher likelihood of developing T2DM by 19% and 40% per 10 μg/m^3^ increase in NO_2_ and PM_10_, respectively. The effect of NO_2_ was rendered nonsignificant in the PM_10_ and NO_2_ model [123]. Another study in China found higher T2DM prevalence to be related to greater PM_2.5_ levels with higher rates among males, less educated individuals, unclean energy users, rural area dwellers, current smokers and those with greater BMI [124]. Two ecological studies also suggest higher PM_2.5_ levels to be associated with higher T2DM rates [78,125]. Several studies nonetheless found no significant or strong association for NO_x_ [87], NO_2_ [86,87,118], PM_10_ [87,126], PM_2.5_ [87,114], lower air quality [127], SO_2_ (sulphur dioxide) [128], and benzo alpha pyrene [114]. One study demonstrated back extrapolated pollutant levels of NO_2_ and NO_x_ to be significantly associated with increased T2DM risk [87].

Studies conducted among women [81,82,84] and African-American women [79,83] showed significant association. Stronger effect of pollution was found among women than men [116,118,122,126,129], and among women living closer to a major road [82,119], and with low education [82]. The association also appeared to be stronger among those with chronic obstructive pulmonary disease [122], non-smokers, physically active individuals, low education, greater waist to hip ratio [116], higher BMI [120], and <50 years and >65 years [116,120,122]. One study found stronger effect among those with higher education levels [120]. Area-level SES [88] and BMI [119] weakened the relationship, and area SES also seemingly masked the impact of PM_2.5_ [89]. In a few studies, the influence of comorbidities and sociodemographic factors [122], area SES, BMI, age, education, exercise level, smoking, hypertension, and diet [88] was not apparent. Most of the studies did not have information on indoor exposure and exposure outside of neighbourhood, and none of the studies used air toxicant levels in blood or biological samples.

### 7.7. Neighbourhood Conditions, Safety, and Other Environmental Characteristics

A cohort study conducted among African-Americans revealed a higher T2DM risk among those living in housing condition rated as fair-poor compared to good-excellent [80]. The relationship was but rendered non-significant when adjusted for psychosocial and health factors, and was not affected considerably when accounted for residential mobility and ownership. In addition, the T2DM prevalence was found to be significantly lower in areas with higher home value in an ecological study [76]. On the other hand, two studies did not find a strong effect of neighbourhood conditions [77,80], and a few studies did not find significant association for perceived neighbourhood safety, crime and physical disorder [77,100,105]. A recent cohort study reported a non-significant elevated T2DM risk with increase in neighbourhood violence and problems [73]. 

Two studies did not find significant difference in the risk of T2DM by availability and type of public open space [91,105], although the study by Paquet et al. [91] suggests a protective effect of greater open space size on T2DM risk. A cross-sectional study that used GIS measure found a lower probability of self-reported T2DM to be associated with higher levels of mean slope [130]. The influence of self-selection and other confounding factors however cannot be ruled out in this study. With regard to health service accessibility, an ecological study suggests no strong association between availability of general practitioners and T2DM [131].

## 8. Discussion

Evidence on the effects of the environment on T2DM risk in adults has grown significantly over the past decade. A majority of studies come from high-income countries and were observational in design. The most common environmental characteristics studied in relation to T2DM were air pollution, walkability, food environment, physical activity resources, and roadways proximity. Overall, the findings in the studies reviewed show moderate evidence of the association between environment and T2DM risk. Living in neighbourhoods with higher levels of walkability and green space was associated with lower T2DM risk, while higher levels of air pollution and noise were associated with increased T2DM risk. There were insufficient data to deduce causal inference between these environmental characteristics and T2DM. Further, the evidence on the role of other characteristics on T2DM is less clear and/or limited. The methodological shortcomings could have led to the inconsistent findings. The results, however, provide enough cause to further delve into understanding the environment–T2DM relationship.

The current evidence is not suggestive of environmental characteristics that may be most significant in T2DM prevention and amenable to policy interventions. The assessment of either one or limited characteristics in the studies reviewed could have resulted in over- or under-estimation of potential impacts. Studies conducted among minority ethnic groups and women indicate a stronger association. Minor ethnic and racial groups are often deprived of recreational facilities, supportive aesthetics and do not have adequate traffic protection and active transportation increasing their susceptibility to adverse health outcomes [5,25,32]. They may also live in neighbourhoods with low SES and a higher density of unhealthy food choices and outlets that promote unhealthy food [5,107,132,133]. 

### 8.1. Walkability

A majority of the studies point that living in walkable neighbourhoods is linked with a lower T2DM risk. This is consistent with the findings of recent systematic reviews that showed walkable environment to be associated with a lower risk of T2DM, metabolic syndrome, obesity, blood pressure [5,20,21] and physical activity [21,134]. However, the underlying factors contributing to this relationship are less clear, and the current data are limited to derive causal inference although the influence of obesity, physical activity, and income has been noted [85,92,95]. Many studies did not consider that those living in a more walkable neighbourhood may be healthier and physically active and did not adjust for sociodemographic factors, safety, crime, traffic, pollution, and other environmental factors [85,91,92,94,95,96].

Assessing the impact of individual walkability components will be essential to identify specific areas for intervention. When evaluated by individual components, only land use mix was found to be significantly associated with T2DM in one study [95], while another found all elements significant [94]. Further, the lower T2DM risk was found to be significantly related to 800 m walkability buffer area but not for 1600 m [92]. Whereas one study showed the effect to be significant for 1600 m buffer area [99], another found similar result for one- and three-mile buffer areas [73]. The area size at which the environment impacts obesity has also been shown to vary for different characteristics [135]. The exact neighbourhood buffer area may vary for different characteristics and may also vary between countries and regions, owing to climatic and cultural factors, and this warrants further investigation.

### 8.2. Physical Activity Resources

The evidence although indicative of lower T2DM rates in areas with more physical activity resources is limited to draw solid inferences. Literature suggests that accessibility to physical activity environment and resources is associated with hypertension, physical activity, obesity and cardiovascular outcomes [5,20,22,40]. Hence, the relationship between T2DM and physical activity resources is plausible. Some studies reviewed interviewed participants to assess resource availability and metrics used varied between studies. The influence of other factors such as safety, food environment and car ownership is also not adequately known. 

The effect of survey-based measure of physical activity resources was found stronger than GIS-based measure [100] suggesting that just having resources may not be enough to enable healthy behaviours. For instance, proximity to open spaces may not be sufficient to motivate walking [33] and opening supermarkets in areas considered “food desert” may not influence diet and BMI [136]. An understanding of the use, quality, and size of these resources, accessibility, and characteristics of those using these resources are essential. 

### 8.3. Food Environment

The current data on the impact of the food environment on T2DM have produced inconsistent results. The heterogeneity across studies concerning food environment and outcome measures and neighbourhood definition could have led to the mixed findings. Literature also suggests a mixed if not negligible effect of the food environment on obesity [5,53]. Both healthy and unhealthy foods can be available in food outlets such as supermarkets, and some fast-food outlets may offer healthy options. Besides, for some individuals, quality and price can be more important than distance and travel cost [137] implying that proximity may not necessarily denote accessibility. The association can be distorted if these aspects are not considered. 

Similar to physical activity resources studies, studies that used survey-based measures tended to show stronger association [99,100]. Differences in perceived and observed environmental measures have also been noted in association with obesity and physical activity [137,138]. An individual’s behaviour can be more closely linked to their perception of the environment [69]. In addition, factors such as affordability, purchasing and intake behaviour and quality that cannot be measured objectively can be equally important. Assessing access to farmer’s and fruit and vegetable markets, specific food types, and considering the influence of marketing and confounders in future studies will enable generation of robust evidence. 

### 8.4. Green Space

The benefit of green space on health is increasingly being recognised [139,140]. This review found that those living in greener neighbourhood have a lower risk of T2DM. A few reviews also portray green space to be protective against obesity related outcomes [140,141]. The current data, however, are limited by design and the green space–T2DM pathway is not adequately studied. For example, whether greenery reduces depressive symptoms [46], promotes walking [27], and/or moderates pollution [52] is not assessed. Future studies should, therefore, investigate the possible mediating and moderating factors. The protective effect of green space also needs to be further corroborated in longitudinal studies. Subsequently, it may be useful to determine the minimal green space level and quality to prevent T2DM. Besides, using distance to green space may be an appropriate measure since the use could decrease with increasing distance [27]. This, however, merits further investigation.

### 8.5. Residential Noise, Traffic, and Proximity to Roads

The evidence on the effect of traffic exposure and proximity to major road is limited and mixed. A recent meta-analysis showed that residential proximity to major road is associated with higher T2DM risk [142]. It is important to note that the current review included a few additional recent studies that found no strong association. The use of subjective measures, small sample, non-random sampling, and inadequate confounder adjustment could have biased the results in some studies. Thus, studies with better designs, adequate sample size using objective measures and accounting for confounders are required. Exposure at the workplace and the use of air and noise pollution measure can help reduce exposure characterization errors. 

On the other hand, the current evidence consistent with the findings in a recent meta-analysis [143] and a review [144] is suggestive of a greater T2DM risk with higher traffic noise levels. The underlying mechanism is nonetheless less clear. Higher noise levels have been associated with increased blood pressure [6]. A stronger effect was observed among those who reported poor sleep quality [113], and sleep is linked with T2DM [144,145]. Hormonal changes due to sleep deprivation can increase appetite that can promote the development of obesity and changes in metabolic functions including elevated blood sugar levels [143,145]. Noise can also increase cortisol levels (a hormone that regulates metabolism and helps control blood sugar levels) and lead to insulin resistance [112]. Further, noise is related to stress [146] and stress can heighten T2DM risk [144]. Chronic stress results in impaired metabolic function and obesity through dysfunction of the hypothalamic-pituitary-adrenal axis, a system that controls stress and body processes [48,147].

The data also suggest that the effect of noise differ by gender, physical activity and education levels [71,112]. Individuals with low education may live in poor housing conditions increasing their vulnerability to noise and related health impacts. Likewise, being physically active may buffer the effect of stress induced by noise. The weak effect of aircraft and railway noise on T2DM found in a few studies [71,112,113] is plausible given the likely low exposure duration and the confinement of exposure to only certain areas. Some studies showed the effect of noise seemingly stronger than air pollution and traffic exposure [112,115,123]. More studies using longitudinal designs can help corroborate the noise–T2DM link, and understand how noise, pollution, and other factors interact to influence T2DM. 

### 8.6. Air Pollution

The evidence though modest is suggestive of a higher T2DM risk with exposure to higher air pollution levels, in particular of NO_2_ and PM_2.5_. This is consistent with findings from recent reviews and meta-analyses [144,148,149,150]. The risk though minimal can translate into a higher proportion of the population impacted given the huge number of people likely to be exposed to pollution. The literature informs that air pollutants can influence T2DM risk through endothelial dysfunction, adipose inflammation and can also trigger insulin resistance [49]. Air pollution has also been associated with hypertension and obesity [6,151]. The data on other pollutants are minimal.

The effect of NO_2_ appeared to be stronger than PM_2.5_ and other pollutants. A recent meta-analysis also showed an enhanced association for gaseous pollutants than particulate matters [148]. Only a few studies used multi-pollutant models, and it will be important to consider all potential sources and type of pollutants to identify the key pollutant. Studies also showed women to be more susceptible from exposure to pollution. It is posited that women spend much of their time in and around the home, tend to choose work nearer home, and may limit work outside the home [118,129]. This can enable precise measurement of exposure at their residential address, hence the increased risk than men. Besides, gender physiological differences may also contribute to this difference [129].

The moderate effect of air pollution on T2DM can also be attributable to the rapid decline in pollution in developed countries [152], and almost all (>90%) studies were from developed countries. Back-extrapolated pollution levels were found to be significantly associated with heightened T2DM risk exhibiting the effect of higher pollution levels [87]. The impact of air pollution and related environmental characteristics is more likely to be stronger in developing countries considering the greater levels of air pollution [152] and the higher T2DM burden [3]. Thus, more studies from developing countries are essential to inform public health decisions. Most studies did not include information on indoor pollution and environmental tobacco smoke, and the effect of other pollutants such as carbon monoxide is not assessed. Besides, exposure outside the neighbourhood such as at workplace, comorbidities, and uncontrolled factors could have influenced the results. Considering these caveats and using time-varying pollution levels in future studies can help produce robust data. 

### 8.7. Neighbourhood Conditions, Safety, and Other Environmental Characteristics

Only a very few studies have assessed the impact of neighbourhood safety, crime, physical disorder, open space, urban sprawl and health accessibility on T2DM. Studies showed that better housing conditions and higher home value [76,80] and urban compactness [97] to be a significant determinant of T2DM. Housing and neighbourhood conditions may influence T2DM risk through mental health [153,154], safety [155], and socioeconomic factors. Neighbourhood safety, physical conditions, and disorder have been associated with physical activity and obesity [44,137,156]. A higher property value can also influence physical activity and healthy diet [157]. For instance, higher real estate value may encompass other characteristics such as open space, aesthetic environment, green spaces and proximity to amenities. Likewise, urban compactness has been associated with lower rates of obesity and hypertension and increased physical activity [158]. A compact neighbourhood can bring amenities and services closer to homes, thus promoting active transportation. Certainly, more research is needed to understand the relationship between these characteristics and T2DM.

Studies assessing the impact of geographic characteristics on T2DM are also limited. A study showed higher levels of slope to be protective against T2DM [130]. Availability of hills in the neighbourhood has been related to higher physical activity levels [30]. Hilly localities may have more pleasant sceneries that could motivate physical activity. In contrast, another study showed that steep hills prevented walking [31]. Cohort studies considering self-selection are essential to substantiate the findings. 

### 8.8. Strengths and Limitations 

To our knowledge, this is the first systematic review investigating the impact of environment on T2DM. The findings are presented and discussed by different environmental characteristics. This work can be, therefore, a good reference for works on the environment and T2DM and related health outcomes. The rigorous peer review and the application of a systematic method to conduct the systematic review in compliance with Preferred Reporting Items for Systematic Reviews and Meta-Analyses (PRISMA) guidelines also lends to its credibility. A preliminary search since 1990 and screening of references of included studies allowed a comprehensive search.

There are some limitations. Firstly, many of the studies were cross-sectional and are inclined to biases outlined in the methodological issues section. Thus, inferences regarding causality cannot be made even for those environmental characteristics suggestive of being predictors of T2DM. Second, this review could have also missed articles published in languages other than English, although it may be reasonable to assume that the numbers will be small. Furthermore, there were only a few studies from developing countries. Thus, findings may not be widely applicable to developing countries. Our review also included studies that also had participants younger than 18 years and many studies that included individuals <18 years and young adults tended to show no strong or null association. 

Information and selection bias, residual confounding from mismeasurement and/or imprecise exposure measurement cannot be excluded in many studies. Additionally, differences in exposure and outcome assessment and the adjustment of factors across studies within particular environmental characteristics could have resulted in the mixed findings and also impeded comparison across studies. Given the high diversity between studies including the effect estimates and the likely different mechanisms involved even within specific characteristics, a meta-analysis was not possible to estimate the pooled effect of environment on T2DM risk. Likewise, the risk of publication bias could not be examined though likely to be negligible given the inconsistent or null associations shown by the studies assessed.

### 8.9. Future Research Directions

More studies with better designs and methods are required to corroborate the current evidence and to understand the role of environment in T2DM prevention. It is likely that the impact of the environment will be pronounced with larger cumulative exposure over time. Therefore, longitudinal studies are likely to detect the impact and determine the time frame for environmental characteristics to influence T2DM. Studies aimed to address issues surrounding environment metrics, and other methodological caveats innate to observational studies are needed. Future studies would also need to use both perceived and objective measures to explain varying associations between the two, and also the policy interventions for the two can be different. Reducing confounding by self-selection, and accounting for changes in environment over time using longitudinal data can help assess causal relationship. Likewise, identifying the point in the lifetime where the environment may have the greatest impact on T2DM using life course data will be beneficial. Further, assessing multiple related environmental determinants will enable identification of the key determinant of T2DM prevention, and thus beneficial in informing policy decisions.

Studies need to assess whether socioeconomic, demographic, psychosocial and behavioural factors influence the environment–T2DM relationship. The interaction between environmental characteristics and other factors also needs to be examined, for example, between noise and air pollution, between pollution and walking environment, and between street networks and food environment and safety. Similarly, the mechanisms by which environment exerts influence on T2DM—for better and for worse—needs to be more clearly elucidated. Another opportunity would be to investigate whether neighbourhood-based policy interventions to improve local environments have yielded a decrease in T2DM burden. Clearly, there is an urgent need for research from developing countries and among vulnerable and rural populations. 

Alongside increasing evidence, future studies should also consider identifying the minimum level and/or threshold and combination of relevant environmental characteristics at which T2DM can be possibly prevented [109]. Likewise, identifying environmental determinants of prediabetes can also inform prevention strategies. Qualitative studies to understand how individuals relate to and interact with the environment can enable generation of better theories on the environment–T2DM pathways [54]. Lastly, more research is needed on other understudied characteristics in the context of T2DM, such as green space, urban sprawl, health service accessibility, neighbourhood conditions and public transport.

## 9. Conclusions

Overall, the current evidence suggests a moderate contribution of environment on T2DM risk. This review nevertheless highlights the potential barriers brought by the environment in reducing T2DM burden through individual-level interventions. The data show that higher levels of walkability and green space are associated with a lower risk of T2DM, while higher levels of NO_2_, PM_2.5_, and noise are related to elevated T2DM risk. However, owing to the limited data on these characteristics, causality cannot be deduced. The existing data on food environment, physical activity resources, traffic, and proximity to major roads are mixed. Likewise, there is a paucity of literature on other environmental characteristics. Finally, the mechanisms through which the environment influences T2DM risk is less clear. A better understanding of the environment–T2DM relationship can inform the formulation of policies that promote health and create opportunities for individuals to translate intentions into sustained behavioural change that are essential to curb the rising burden of T2DM. 

## Figures and Tables

**Table 1:** Terms used to search relevant literature.

Sl	Search Terms
1	type 2 diabetes OR non-insulin-dependent diabetes OR prediabetes OR diabetes mellitus
2	1 AND built environment OR neighbo?hood environment
3	1 AND walkability OR green space OR greenspace OR parks OR open space OR trees OR land use mix
4	1 AND food environment OR supermarket OR fast food outlet OR cafe OR bar OR liquor store OR restaurant
5	1 AND public transport OR street connectivity OR road traffic OR train OR bus
6	1 AND air pollution OR noise pollution
7	1 AND neighbo?hood safety OR neighbo?hood crime
8	1 AND amenit * OR recreational facility *
9	1 AND access to primary health care OR health care accessibility OR access to health care OR availability of health service OR availability of health care OR health facility density OR proximity to health facility

* ?: truncation symbols used to enable search.

**Table 2:** Summary of studies reviewed.

Characteristics	Categories	Number ^i^
Study year (publication)	2005–2006	1
	2007–2008	2
	2009–2010	5
	2011–2012	7
	2013–2014	17
	2015–2017	28
Study design	Cohort/longitudinal	26
	Cross-sectional	24
	Ecological	10
Country	USA	24
	Canada	6
	Germany	5
	Australia	5
	Others ^ii^	20
Country income level	High income country	56
	Upper middle income country	4
Environment focus	Distance to roadways	7
	Food environment	17
	Physical activity resources	8
	Walkability ^iii^	7
	Neighbourhood conditions ^iv^	4
	Crime/physical disorder/safety	4
	Green space/tree canopy	4
	Open space	2
	Others ^v^	4
	Air pollution/quality	25
	Noise pollution	4
Air pollutants *	PM_2.5_ (particulate matter of <2.5 µm)	14
	NO_2_ (nitrogen dioxide)	11
	PM_10_ (particulate matter of <10 µm)	8
	NO_x_ (nitrogen oxides)	5
	SO_2_ (sulphur dioxide)	2
	PM_10–2.5_ (particulate matter of 2.5–10 µm)	2
	BaP (Benzo alpha pyrene)	1
	Ozone	1
	Soot	1
	Air quality	1
Noise pollution source *	Traffic noise	3
	Railway noise	2
	Aircraft noise	2
Environment measurement	Objectively measured	51
	Reported by study participants/surveys	3
	Survey and objective measures combined	6
Outcome	Type 2 diabetes mellitus	25
	Diabetes Mellitus	35
	Prediabetes and diabetes mellitus	4
Outcome assessment	Self-reported ^vi^	25
	Blood sugar tests ^vii^	13
	Database/registers/records	22
Association	Significant in expected direction	82
	Non-significant in expected direction	81
	Non-significant in unexpected direction	33
	Null association	6
Study quality ^viii^	Good	11
	Fair	32
	Poor	17

^i^ study may be counted more than once since several environment characteristics were assessed in some studies; ^ii^ includes UK, Netherlands, Sweden, Korea, Denmark, Switzerland, China, Bulgaria, Iran, and Jamaica; ^iii^ also includes studies that assessed walkable destinations; ^iv^ one each of neighbourhood and housing conditions, infrastructure, and home value; ^v^ include urban sprawl, area level slope, natural amenities and general practitioners; ^vi^ combination of self-reported and blood sugar tests are included under blood sugar tests; ^vii^ blood sugar tests also include HbA1c; ^viii^ study quality was assessed using the National Institutes of Health’s quality assessment tool for observational cohort and cross-sectional studies [70]; * sub-components under the broader characteristics “Environment focus”.

## Supplementary Materials

The following are available online at www.mdpi.com/1660-4601/15/1/78/s1, Table S1: Key characteristics of studies included in the systematic review.
