# Supplementary material for: Environmental Risk Factors for Developing Type 2 Diabetes Mellitus: A Systematic Review
Source: Int J Environ Res Public Health. 2018 Jan 5;15(1):78. doi: 10.3390/ijerph15010078 (PMC5800177; doi:10.3390/ijerph15010078)
Supplement: Supplementary file 1 [file ijerph-15-00078-s001.pdf]

Supplementary Table

Table S1: Key characteristics of studies included in the systematic review

**Author # / (year)**	**Country/ (income level)**	**Study design**	**No. of participants/ Sex**	**Age (years)**	**Environment focus**	**Exposure assessment**	**Outcome assessment**	**Effect size/ association (95% CIs)**	**Factors adjusted**	**Study quality rating**
Bodicoat, (2014) [110]	United Kingdom (HIC)	CSS	10476 / men and women	25-75	Green space	GIS	Blood test*	OR=0.45 (0.24-0.82), highest vs lowest quartile.	Age, sex, ethnicity, rural/urban, area social deprivation, BMI, ethnicity, objective PA, cholesterol	Fair
Maas, (2009) [72]	Netherlands (HIC)	CSS	343103/ men and children	< 12-65+	Green space	Land cover database	Medical records	OR=0.98 (0.97-0.99), 0.98 (0.97-1.00) with 10% more than average greenspace within 1 km and 3 km radius, respectively.	Age, sex, work status, education, health insurance, urbanicity	Fair
Astell-Burt, (2014) [109]	Australia (HIC)	CSS	267072/ men and women	45+	Green space	GIS	Self-reported	OR=0.99 (0.96-1.03), 0.90 (0.85-0.96), 0.90 (0.85-0.96), 0.91 (0.84-0.99), 0.94 (0.85-1.03) for 21-40%, 41-60%, 61-80%, >80% of greenspace, compared to <21% greenspace.	Age, sex, marital status, ancestry, birth country, language, weight, distress risk, smoking status, hypertensive, diet, PA, employment, education, income, urban/rural, area deprivation and remoteness	Fair
Ulmer, (2016) [111]	USA (HIC)	CSS	4820/ men and women	46 (mean age), adults of < 65	Tree cover	High-resolution imagery, remote sensing	Self-reported	OR=0.81 (0.64-1.03) for 10% increase in tree canopy coverage.	Age, gender, ethnicity, marital status, education, employment, English proficiency, food security, income, home ownership, duration at current address, smoking, insurance, household size, poverty and survey cycle, walkability	Fair

Auchincloss, (2009) [99]	USA (HIC)	CS	2285/ men and women	45-84	PA and food environment	Telephone survey	Blood test/ insulin use/ medication use / self-reported	HR=0.64 (0.44-0.95) for PA and healthy food combined.<br>HR=0.71 (0.48-1.05) and 0.63 (0.42-0.93) for PA and healthy food resources respectively, difference between the 90th and 10th percentiles.	Age, sex , ethnicity, household income and assets, education, smoking status, alcohol use, family history of diabetes, BMI, PA, diet	Good
Christine, (2015) [100]	USA (HIC)	CS	5124 / men and women	45-84	PA and food environment, safety	GIS, survey	Blood test/ insulin use/ medication use	HR=0.93 (0.82-1.06), 0.81 (0.68-0.96), 0.98 (0.88-1.10) for healthy food, PA and social environment respectively, corresponds to an IQR increase.	Age, sex, income, family history of T2DM, stress, educational level, race/ethnicity, alcohol use, smoking, BMI, diet, PA, neighbourhood SES	Good
Gebreab, (2017) [73]	USA (HIC)	CS	3670 / African-American men and women	21-94	PA, food environment	GIS, survey	Self-reported/ blood test/ medication use	HR=1.07(0.87-1.32) for violence, and 1.15(0.92-1.44) for problems, difference between 90th and 10th percentiles.<br>HR=1.23(0.98-1.55), 1.34(1.12-1.650, and 1.11(0.97-1.26) for favourable food, unfavourable food and PA resources respectively.	Age, gender, family diabetes history, BMI, physical activity and diet	Fair
Polsky, (2016) [74]	Canada (HIC)	CS	7079 / men and women	20-84	Fast food restaurants (FFRs)	ArcGIS, database	Database	HR=1.27 (0.78-2.06), corresponds to one IQR increase in percentage of FFRs.<br>HR=0.95 (0.87-1.04) and 0.92 (0.84-1.01) for absolute FFRs and total restaurants respectively.	Sex, age, ethnicity, immigration status, education, income, smoking, residence city, area disadvantage, walkability, survey cycle, BMI	Fair
Mezuk, (2016) [103]	Sweden (HIC)	CS	4718583 (varied for specific analysis) / men and women	35-80	Food environment	GIS	Medical records	OR=2.11 (1.57-2.82) for incident, 1.85 (1.51-2.26) for prevalent cases.<br>OR=3.67 (2.14-6.30) among those locating to areas with more health harming food,	Age, sex, education, income, neighbourhood deprivation	Fair

								1.72 (1.27-2.33) among those who did not move but gained access to more health harming food outlets.		
Piccolo, (2015) [105]	USA (HIC)	CSS	2746/ men and women (three ethnic groups)	30-79	Food environment, open space, crime/ disorder	GIS, police department website, food establishment data, interviews	Self-reported/ blood test	OR=0.53(0.25-1.15), for those living a mile farther from a grocery store.	Race, gender, age, income, education, PA, BMI, poverty	Fair
Bodicoat, (2014) [107]	United Kingdon (HIC)	CSS	10461/ men and women	18-75	Food environment	Online business listings	Screen-detected	RR=1.02 (1.00-1.04), (p=0.02) for every increase in fast-food outlet number.	Social disadvantage, age, sex, rural/urban, ethnicity	Poor
Morland, (2006) [106]	USA (HIC)	CSS	10763/ men and women	49-73	Food environment	Agriculture and environmental health data, GIS	Self-reported on anti-diabetics/ blood test	PR=0.96 (0.84-1.10), 1.11 (0.99-1.24), 0.98 (0.86–1.12) lived in areas with supermarkets, grocery stores and convenience stores respectively.	Age, income, sex, race, PA, education, types of food stores and service places	Poor
Frankenfeld, (2015) [104]	USA (HIC)	ES	3227 blocks	> 18	Food environment	GIS, database	Self-reported	Significantly lower diabetes prevalence in food source subtypes with greater restaurant and speciality food than those with grocery store within the healthier options category.	Neighbourhood SES and demographic factors	Poor
Auchincloss, (2008) [98]	USA (HIC)	CSS	2026/ men and women	45-84	PA and food environment	Survey	Blood test/ diabetes treatment	Decrease by 6% (-30%-27%), increase by 22% (-10%-64%), 14% (2%-27%) and 3% (-7%-14%), for PA resources, healthy food resources, distance to PA resources and distance to healthy food resources, respectively, corresponding to difference between 90th and 10th percentile.	Age, sex, family diabetes history, income and education, ethnicity, PA, diet, BMI	Fair
Stewart, (2011) [75]	USA (HIC)	ES	442830/ men and women	> 18	Food environment	Business directory, GIS	Database	OR=0.4 (0.09-1.41) and 1.5 (0.41-5.63) for per capita FFRs and convenience stores respectively	Area-level SES, rurality, unemployment index	Poor
Drewnowski,	USA (HIC)	ES	59767 (371	> 18	Food	Food environment	Database	β= - 0.6 (-0.9 to -0.2) for	Age, ethnicity,	Poor

(2014) [76]			census tracts) / men and women		environment, home value	index		each 50% increase in median home value. No association for retail food environment.	population density, area-based socioeconomic variables, obesity	
Salois, (2012) [102]	USA (HIC)	ES	3051 counties	> 20	Food environment, recreational and natural amenities	Food environment atlas	Self-reported	Significant positive association with diabetes for % of household without car and >1 mile to store, % low income and >1mile to store, fast-food restaurant density, convenience stores without gas density. Significant negative association for full-service restaurant and farmer's market density. Other characteristics not significant.	Ethnicity, income, poverty rate	Poor
Alhasan, (2016) [108]	USA (HIC)	ES	46 counties/ men and women	> 20	Food environment	Agriculture database	Institutional records	β= -0.55 (p=0.54), 0.89 (p=0.31), -0.40 (p=0.97), -3.70 (p=0.09) for density of fast food restaurants, convenience stores, super stores and grocery stores respectively.	County level PA, obesity, recreational facility, education, unemployment, access to stores, household car ownership and race/ethnicity	Fair
Ahern, (2011) [101]	USA (HIC)	ES	3128 counties/ men and women	> 20	Food and recreational environment	Food environment atlas	Database	β=0.07 (p=0.01), 0.41 (p=0.07), -0.15 (p=0.04), -0.37 (p=0.09), 0.03 (0.06), -0.01 (p=0.004), -0.12 (p=0.21) for % households without car living >1 mile from grocery store, fast-food restaurants per 1000, full service restaurants per 1000, grocery store per 1000, convenience stores per 1000, direct farm sales per capita, and recreational facilities per 1000 respectively.	Sex, poverty, doctors availability, nativity / ethnicity, education, smoking, obesity	Poor
Paquet, (2014) [91]	Australia	CS	3205/ men	≧ 18	Walkability,	ArcGIS, road	Blood test	RR=0.99 (0.90-1.09), 0.88	Age, gender, income,	Fair

	(HIC)		and women		POS, food outlets	network distance, retail database		(0.80-0.97) and 1.00 (0.92-1.08) for one standard deviation increase in relative unhealthy food environment, walkability and POS count respectively. RR=0.75 (0.69-0.83), 1.01 (0.90-1.13), 1.09 (0.97-1.22) for one standard deviation increase in POS size, POS greenness and POS type respectively.	education, follow up duration, neighbourhoods deprivation	
Booth, (2013) [85]	Canada (HIC)	CS	1239262/ men and women	30-64	Walkability	Walkability quintile using GIS	Database	RR=1.58 (1.42-1.75), 1.67 (1.48-1.88) among recent immigrant men and women respectively. RR=1.32 (1.26-1.38), 1.24 (1.18-1.31) among long-term men and women residents respectively, living in least walkable areas.	Age, income (area poverty)	Fair
Sundquist, (2015) [95]	Sweden (HIC)	CS	512061/ men and women	> 18	Walkability	Walkability index	Drug registers	OR=1.33 (1.13-1.55) when adjusted for neighbourhood deprivation, and OR=1.16 (1.00-1.34) upon addition of individual sociodemographic factors. OR=0.97 (0.30-2.46), 1.07 (0.91-1.25), 1.23 (1.07-1.42) for residential density, street connectivity and land use mix respectively, lowest compared to highest deciles of walkability.	Age, gender, income, education, neighbourhood deprivation	Fair
Lee, (2015) [96]	Korea (HIC)	CSS	16178/ men and women	≧ 20	Walkability	GIS, physical observation	Database	OR=0.86 (0.75-0.99) living in more compared to living in less walkable environment.	Smoking, alcohol use, age, sex, income	Fair
Glazier, (2014)	Canada	ES	10180 blocks/	30-64	Walkability,	Walkability index	Database	PRR=1.33 (1.33-1.33), 1.26	Prevalence rate	Poor

[94]	(HIC)		men and women		walkable destinations			(1.26-1.26) for walkability index and destination within 800m, respectively for the lowest compared to highest quintile. All individual walkability components were significant.	adjusted for area-level age and sex	
Müller-Riemenschneider, (2013) [92]	Australia (HIC)	CSS	5970 /men and women	≧ 25	Walkability	Walkability index using GIS	Self-reported	OR=0.79 (0.52-1.21) among participant of most walkable areas. Significant when not adjusted for PA and sedentary behaviour	Age, sex, income, marital status, education, physical activity, sedentary behaviour	Fair
Creatore, (2016) [93]	Canada (HIC)	CS	>2770000/ men and women	30-64	Walkability	Walkability index using GIS	Database	Incidence lower by 1.7 per 1000 persons (CI -2.8 to -0.7) in the highest compared to lowest walkability areas.	Neighbourhood age difference, sex, income, ethnicity	Fair
Cunningham-Myrie, (2015) [77]	Jamaica (UMIC)	CSS	2848/ men and women	15-74	Area infrastructure, recreational space, walking distance, safety	Interviewer assessment	Self-reported / blood test	OR=1.02 (0.95-1.10), 1.12 (0.86-1.45), 1.01 (0.77-1.32), 0.99 (0.88-1.11) and 0.99 (0.95-1.03) for increased levels of infrastructure, recreational in walking distance, recreational space, safety perception, and area disorder, respectively.	Age, sex, fruit and vegetable consumption	Poor
Schootman, (2007) [80]	USA (HIC)	CS	644/ men and women (Africa-American)	56.2 (mean age)	Area and housing conditions	Physical observation, survey	Self-reported	OR=1.85 (1.04-3.30) and 2.12 (1.12-4.02) for adverse housing conditions adjusted for behaviour and sociodemographic factors respectively. No association for neighbourhood conditions.	Age, gender, income, education, marital status, employment, duration of stay at current address, home ownership, sampling strata	Good
Ewing, (2014) [97]	USA (HIC)	ES	Different sample sizes	≧ 30	Urban sprawl	Compactness index using GIS	Self-reported	β = -0.0015 and -0.0016, (p=0.05) for original and refined compactness index, respectively.	Sex, age, race, income, education, smoking, fruits and vegetables consumption	Poor
Villanueva, (2013) [130]	Australia (HIC)	CSS	15954/ men and women	≧ 25	Slope	GIS	Self-reported	OR=0.72 (0.55-0.95), 0.52 (0.39-0.69) living in moderate and higher levels	Age, sex, education, income, PA, diet, and destination to parks,	Fair

								of slope, respectively.<br>OR=0.87 (0.80-0.94) for each increase in a % mean slope.	retail, health services, recreation, fast food/takeaway, larger food outlets, restaurants /cafes /coffee, other food	
Kauhl, (2016) [131]	Germany (HIC)	ES	1.79 million/ men and women	All ages	General practitioners	Inhabitants to practitioner ratio, street maps	Database	No association (effect size not provided).	Not reported.	Poor
Heidemann, (2014) [115]	Germany (HIC)	CS	3604/ men and women	18-79	Traffic	Self-reported	Self-reported/ on anti-diabetic drugs/ insulin	OR=1.15 (0.80-1.67), 1.11 (0.69-1.80), 1.41 (0.96-2.08), and 1.97 (1.07-3.64) for moderate, considerable, heavy and extreme respectively than no/very rare traffic.	Sex, age, smoking, passive smoking, heating of house, education, waist size, sports, parental history of diabetes	Fair
Ward-Caviness, (2015) [117]	USA (HIC)	CSS	2124 / men and women	61.3 (mean age) (adults)	Traffic, road proximity	GIS	Blood test	OR=1.00 (0.88-1.13) for an IQR decrease in the distance to roadways. No or no strong association by traffic exposure zones.	Ethnicity, sex, BMI, smoking, home value	Fair
Sorensen, (2013) [112]	Denmark (HIC)	CS	53673/ men and women	50-64	Traffic, railway noise	SoundPLAN, Nordic prediction method using GIS	National registry	IRR=1.08 (1.02-1.14) and 1.11 (1.05-1.18) for a 10 dB higher level of road traffic noise at current residence and during 5 years preceding diagnosis for all diabetes.<br>IRR=0.97 (0.89-1.05) and 1.01 (0.91-1.11), for exposure to railway noise of $\geq$ 60 dB among all and confirmed diabetes, respectively.	Age, sex, BMI, waist size, smoking, environmental tobacco smoke, saturated fat intake, alcohol, sports, bicycling, walking, fruits and vegetables, air pollution, lifestyle education, occupation, area SES, NO_x exposure	Good
Eriksson, (2014) [71]	Sweden (HIC)	CS	5156/ men and women	35-56	Aircraft noise	GIS	Blood test	OR=0.91 (0.78-1.04), 1.03 (0.84-1.26) for prediabetes and T2DM respectively, for a unit increase in noise.	Sex, age, family history, SES, PA, tobacco, distress, mean income, unemployment rate	Fair
Eze, (2017) [113]	Switzerland (HIC)	CS	2363/ men and women	Adults		Noise emission and propagation models, dispersion	Blood test/ self-reported on	RR=1.35 (1.02-1.78), 1.87 (0.96-3.62), 0.94 (0.71-1.24), 0.87 (0.60-1.22) for per IQR	Ag, sex, education, area SES, income, crowding, mean	Fair

						and land use regression models	medication	increase in day-evening-night road noise, aircraft noise, railway noise and NO_2 respectively. HR=2.08 (1.06-4.08) among those in highest noise level quartile. No strong associations with $PM_{2.5}$.	household rent, smoking, passive-smoke exposure, alcohol, fruits and vegetable consumption, PA, BMI, noise annoyance, green areas	
Dzhambov, (2016) [114]	Bulgaria (HIC)	CSS	513/ men and women	> 18	Noise, $PM_{2.5}$, BaP (benzo alpha pyrene), traffic	Dispersion models, traffic noise maps and surveys	Self-reported	OR=4.49 (1.38-14.68) for day-evening-night equivalent sound of 71-80 dB compared to 51-70 dB. OR=1.32 (0.28-6.24) for $PM_{2.5}$ 25.0-66.8 μg/m^3 than 0.0-25.0 μg/m^3. OR=1.76 (0.52-5.59) for BaP 6.0-14.02 ng/m^3 compared to 0.0-6.0 ng/m^3. OR=1.40 (0.8-4.07) for high self-reported traffic intensity.	Age, gender, ethnicity, education, marital status, occupation, SES, BMI, PA, diet, alcohol use, smoking, family history of diabetes, duration of stay at current address, bedroom location, sleeping with open windows, sleep disturbance	Fair
Andersen, (2012) [116]	Denmark (HIC)	CS	51818/ men and women	50-65	NO_2, NO_x, road proximity, traffic	AirGIS human exposure modelling	National register	HR=1.00 (0.97-1.03), 1.00 (0.97-1.04), 0.98 (0.95-1.01), 1.00 (0.90-1.11), 1.00 (0.98-1.02), 1.01 (0.98-1.03) for an IQR increase in NO_2 1971 to follow-up, NO_2 1991 to follow-up, NO_2 at baseline, major road within 50m, traffic load within 100m and NO_2 at follow-up respectively, among all diabetes cases. HR=1.04 (1.00-1.08), 1.04 (1.01-1.07), 1.02 (0.98-1.05), 1.07 (0.95-1.21), 1.02 (1.00-1.04), 1.04 (1.01-1.07) corresponding effect among confirmed cases. Similar but weaker associations for NO_x.	Age, sex, BMI, waist-to-hip ratio, smoking, environmental tobacco smoke, education, PA, alcohol intake, fruit and fat intake, calendar year	Good

Coogan, (2012) [83]	USA (HIC)	CS	3992/ women (African origin)	21-69	$PM_{2.5}$, NO_x	Kriging for $PM_{2.5}$ and land use regression model for NO_x	Self-reported	IRR=1.15 (0.51-2.58), 1.24 (1.05-1.45), corresponding to 10 unit $PM_{2.5}$ increase and IQR increase of NO_x respectively.	Age, BMI, education, income, number of household members, smoking, drinking, PA, neighbourhood SES, family history of diabetes	Fair
Coogan, (2016) [88]	USA (HIC)	CS	43003/ women (African origin)	≧ 30	NO_2	Land use regression model and dispersion model	Self-reported	HR=0.96 (0.88-1.06), 0.94 (0.80-1.10) using land use regression and dispersion model respectively, corresponds to per IQR increase.	Age, metropolitan area, education, vigorous exercise, BMI, smoking, diet	Fair
Chen, (2013) [122]	Canada (HIC)	CS	60076 / men and women	≥ 35	$PM_{2.5}$	Satellite based estimation using spectroradiometer	Database	HR=1.11 (1.02-1.21) for 10 μg/m^3 increase.	Smoking, sex, marital status, education, household income, BMI, PA, smoking, alcohol, diet, hypertension, urbanicity, race, and area SES, comorbid conditions	Good
Coogan, (2016) [89]	USA (HIC)	CS	43003 / women (African origin)	30-69	$PM_{2.5}$	Land-use regression and Bayesian maximum entropy models	Self-reported	HR=0.99 (0.90-1.09) per 2.9 μg/m^3 increase.	Age, metro area, questionnaire cycle, BMI, area SES, education, exercise, diet	Good
Jerrett, (2017) [79]	USA (HIC)	CS	43003/ women (African-American)	21-69	Ozone	Bayesian space-time model using monitoring data.	Self-reported	HR=1.18 (1.04-1.34) for an IQR increase in O_3. HR=1.20 (1.05-1.37) and 1.13 (0.97-1.31) upon $PM_{2.5}$ and NO_2 adjustment respectively.	Age, period, city, smoking, education, fruit and vegetable, PA, family history of diabetes, BMI, area SES	Fair
To, (2015) [84]	Canada (HIC)	CS	29549/ women	40-59	$PM_{2.5}$	Satellite-based estimates	Database	PRR=1.28 (1.16-1.41), and IRR = 1.28 (1.13-1.45) corresponds to per unit (10 μg/m^3) increase.	Age, education, occupation, marital status, smoking, BMI, mean income, proportion with high school education, low income households, unemployment, smoking, obesity	Fair

Puett, (2011) [119]	USA (HIC)	CS	89460/ men and women	30-55	$PM_{2.5}$, PM_{10}, $PM_{10-2.5}$, road proximity	Spatiotemporal models using monitoring and GIS data, distance using GIS	Self-reported	HR=1.03 (0.96-1.10), 1.04 (0.99-1.09), 1.04 (0.99-1.09) for an IQR increase in $PM_{2.5}$, PM_{10} and $PM_{10-2.5}$ respectively.<br>HR=1.11 (1.01-1.23), 0.96 (0.63-1.48), 0.96 (0.87-1.06) for 0-49m, 50-99m and 100-199m respectively, than ≥200m.<br>HR=1.14 (1.03-1.27) for <50m than ≥200m in women.	Age, season, year, state of residence, time varying covariates (smoking, hypertension, alcohol consumption, diet), PA, BMI	Good
Kramer, (2010) [82]	Germany (HIC)	CS	1775/ women	54-55	PM_{10}, NO_2, Soot	Monitoring station and emission inventory data, land use regression model	Self-reported	HR=1.16 (0.81-1.65), 1.34 (1.02-1.76) for PM_{10} and NO_2 respectively from monitoring stations.<br>HR=1.15 (1.04-1.27), 1.15 (1.04-1.27) for PM and NO_2 from emission inventory.<br>HR=1.27 (1.09-1.48), 1.42 (1.16-1.73) for soot and NO_2 from land use regression (corresponds to per IQR increase).	Age, BMI, fossil fuel heating, work place exposure, temperature, smoking, education	Good
Hansen, (2016) [81]	Denmark (HIC)	CS	24174/ women	> 44	$PM_{2.5}$, PM_{10}, NO_2, NO_x	AirGIS dispersion model	Diabetes register	HR=1.11 (1.02-1.22), 1.06 (0.98-1.14), 1.05 (0.99-1.12), 1.01 (0.98-1.05) per IQR increase in $PM_{2.5}$, PM_{10}, NO_2 and NO_x respectively.<br>HR=1.41 (1.05-1.88), 1.22 (0.93-1.59) per 10 μg/m³ IQR increase in $PM_{2.5}$ and PM_{10}.	Age, calendar year, smoking, PA, alcohol use, fruits and vegetable, fat consumption, employment, marital status, BMI, hypertension, myocardial infarction	Good
Weinmayr, (2015) [120]	Germany (HIC)	CS	3607 / men and women	45-75	$PM_{2.5}$, PM_{10}, road proximity	Dispersion and transport model using emission, meteorology and topography data, GIS	Self-reported/ on medicine/ blood test	RR=1.05 (1.00-1.10), 1.03 (0.95-1.12), 1.36 (0.98-1.89), 1.36 (0.97-1.89) per 1 μg/m³ increase of PM_{10ALL}, $PM_{2.5ALL}$, PM_{10TRA}, $PM_{2.5TRA}$ respectively.<br>RR=1.37 (1.04-1.81), 0.77 (0.57-1.04) for ≤100m and	Age, sex, lifestyle factors, BMI, individual and area SES, city	Fair

								100-200m of distance to major road than >200m.		
Park, (2015) [121]	USA (HIC)	CS	10974/ men and women	45-84	$PM_{2.5}$, NO_x, roadway proximity	Spatiotemporal model based on air quality, land use and traffic data. ArcGIS, road network.	Blood test	OR=1.16 (0.94-1.42), 1.29 (0.94-1.76) for an IQR increase in $PM_{2.5}$ and NO_x respectively. HR = 1.05 (0.87-1.26), 1.04 (0.77-1.40) for incident cases. No significant association for major roadways proximity.	Age, sex, race/ethnicity, family history of diabetes, education, smoking, alcohol consumption, PA, area SES, BMI, study site	Good
Tong, (2015) [128]	China (UMIC)	CSS	~ 77000/ men and women	> 4	PM_{10}, SO_2, NO_2	Environment monitoring website	Database	% diabetes morbidity increase=0.39% (−0.42 to 1.12), 0.15% (−0.25 to 0.54), 1.22% (0.51 to 2.96) for increase in 10 μg/m^3 of PM_{10}, SO_2 and NO_2, respectively.	Age, sex, seasonality, time-varying influences on admission, temperature /humidity, day of the week	Poor
Teichert, (2013) [87]	Germany (HIC)	CSS	363/ women	54-55	NO_X, NO_2, $PM_{2.5absorbance}$, $PM_{2.5}$, PM_{coarse}, PM_{10}	GIS, monitoring station data and land use regression models	Blood test	OR=1.218 (0.909-1.630), 1.224 (0.926-1.617), 1.110 (0.889-1.385), 1.117 (0.808-1.543), 1.075 (0.833-1.388), 1.145 (0.896-1.465) corresponds to an increase in one IQR of NO_2, NO_x, $PM_{2.5absorbance}$, $PM_{2.5}$, PM_{coarse}, PM_{10}, respectively.	Age, BMI, smoking, passive smoking, indoor mould, education and season of blood sampling	Fair
Tahmasebi, (2015) [127]	Iran (UMIC)	CSS	1467/ men and women	Adult	Air quality	ArcGIS	Self-reported	No significant association found. (R^2=0.08).	Not mentioned	Poor
Sohn, (2017) [126]	South Korea (HIC)	CSS	96068/ men and women	46.7 (mean age)	PM_{10}, SO_2	Monitoring station data	Self-reported	OR=1.003 (0.998-1.008), 1.008 (1.003-1.013) with each 1000ppm increase in PM_{10} among males and females respectively. OR=0.979 (0.952-1.006), 1.032 (1.004-1.062) with each 1000ppm increase in SO_2 among males and females respectively.	Age, BMI, household income, economic activity, education, and smoking	Fair
Lazarevic, (2015) [86]	Australia (HIC)	CSS	26991/ women	31-36, 59-64,	NO_2, road proximity	Satellite-based land use regression	Self-reported	RR=1.04 (0.90-1.20), 0.99 (0.95-1.04), 0.98 (0.94-1.02)	Age, BMI, smoking, alcohol consumption,	Fair

				and 85-90		models		corresponding to an IQR NO_2 increase, doubling in the distance to major road and doubling in the distance to minor road, respectively.	PA, fruits and vegetables intake, residential urbanization, temperature, marital status, education, financial resources	
Eze, (2014) [123]	Switzerland (HIC)	CSS	6392/ men and women	29-73	PM_{10}, NO_2	Dispersion models, a hybrid model involving land use regression model.	Self-reported / blood test/ on medication	OR=1.19 (1.03-1.38), 1.40 (1.17-1.67) corresponding to per 10 μg/m^3 increase in NO_2 and PM_{10} respectively, for single pollutant model. OR=1.02 (0.84-1.25), 1.37 (1.02-1.84) for two-pollutant model.	Age, sex, BMI, education, area SES, smoking, environmental tobacco smoke, alcohol use, occupational exposure, fruits and vegetables consumption, noise exposure	Fair
Liu, (2016) [124]	China (UMIC)	CSS	11847/ men and women	≧45	$PM_{2.5}$	ArcGIS, satellite model using spectroradiometer	Blood test/ self-reported	PR=1.14 (1.08-1.20) for an IQR increase in $PM_{2.5}$.	Age, sex, residence location, education, BMI, smoking, alcohol use, heating energy type, ozone	Fair
Dijkema, (2011) [118]	Netherlands (HIC)	CSS	8018/ men and women	50-75	NO_2, road proximity, traffic	Land use regression, GIS	Self-reported/ blood test	OR=0.80 (0.63-1.02), 0.88 (0.70-1.13), 0.96 (0.75-1.22), 1.09 (0.85-1.38) in the highest than lowest quartile of NO_2, distance to main road, traffic flow, traffic within 250m buffer, respectively.	Income, age, gender	Poor
Brook, (2008) [129]	USA (HIC)	CSS	7634/ men and women	≧40	NO_2	ArcGIS	Database	OR=1.04 (1.00-1.08), 0.99 (0.95-1.03), 1.015 (0.98-1.049) for females, males and both sexes respectively, corresponds to increases in over 1 ppb.	Age, BMI, neighbourhood income	Poor
Chien, (2015) [125]	USA (HIC)	ES	3109 counties	>18	$PM_{2.5}$	Air quality monitoring system data	Self-reported	RR=−5.47% (−6.14 - −4.77) to 2.34% (2.0-2.70), increase for every unit increase in $PM_{2.5}$ concentration.	SES variables (male, education, ethnicity family income, occupation, health insurance-all in %),	Poor

									PA, obesity and smoking prevalence, time	
Pearson, (2010) [78]	USA (HIC)	ES	766 counties/ men and women	≧ 20	$PM_{2.5}$	Database and air model data	Self-reported	β=0.78 (0.39-1.25), 0.81 (0.48-1.07) for 2004 and 2005 respectively, corresponds to % increase per 10 μg/m^3.	Age, sex, income, education, ethnicity, health insurance, obesity, PA, latitude, population density	Poor

[#]only the surname of the first author is used in this table, CI: confidence intervals, HIC: high-income country, CSS: cross-sectional study, GIS: geographic information system, *includes all blood test to diagnose T2DM, OR: odds ratio, BMI: body mass index, PA: physical activity, CS: cohort study, HR: hazard ratio, IQR: inter quartile range, T2DM: type 2 diabetes mellitus, SES: socioeconomic status, RR: relative risk, PR: prevalence ratio, ES: ecological study, POS: public open space, PRR: prevalence rate ratio, UMIC: upper middle-income country, IRR: incidence rate ratio, NO: nitrogen oxides, PM: particulate matter of different sizes, SO_2: sulphur dioxide, ppm: parts per million, ppb: parts per billion.
